# Circulating monocytes from prostate cancer patients promote invasion and motility of epithelial cells

**DOI:** 10.1002/cam4.1695

**Published:** 2018-08-09

**Authors:** Karen A. Cavassani, Rebecca J. Meza, David M. Habiel, Jie‐Fu Chen, Alexander Montes, Manisha Tripathi, Gislâine A. Martins, Timothy R. Crother, Sungyong You, Cory M. Hogaboam, Neil Bhowmick, Edwin M. Posadas

**Affiliations:** ^1^ Urologic Oncology Program/Uro‐Oncology Research Laboratories Samuel Oschin Comprehensive Cancer Institute Cedars‐Sinai Medical Center Los Angeles CA 90048 USA; ^2^ Division of Pulmonary and Critical Care Medicine Department of Medicine & Women's Guild Lung Institute Cedars‐Sinai Medical Center Los Angeles CA 90048 USA; ^3^ Department of Medicine Cedars‐Sinai Medical Center Los Angeles CA 90048 USA; ^4^ F. Widjaja Foundation Inflammatory Bowel and Immunobiology Research Institute Cedars‐Sinai Medical Center Los Angeles CA 90048 USA; ^5^ Department of Pediatric, Infectious diseases and Immunology Cedars‐Sinai Medical Center Los Angeles CA 90048 USA; ^6^ Division of Hematology/Oncology Department of Medicine Cedars‐Sinai Medical Center Los Angeles CA 90048 USA; ^7^ Translational Oncology Program Samuel Oschin Comprehensive Cancer Institute Los Angeles CA 90048 USA

**Keywords:** chitinase‐3‐like 1 (YKL‐40), IL‐1β, mCRPC, metastasis, monocytes, prostate cancer

## Abstract

**Background:**

Recruited myeloid cells are known to promote cancer initiation, malignant progression, metastasis, and resistance to therapy in the tumor niche. We tested the hypothesis that circulating blood monocytes from advanced prostate cancer (PCa) patients exhibit a protumor phenotype and directly influence the tumor microenvironment in response to tumor‐derived signals.

**Methods:**

Blood monocytes from advanced and stable PCa patients were cultured, and the conditioned media (CM) were collected and analyzed using standard invasion and wound closure assays to measure effects on invasion and motility of PCa tumor cells. We then identified the proteome profile of these monocytes using proteome array and ELISA.

**Results:**

Conditioned media from circulating monocytes in patients with metastatic prostate cancer (PCa‐M) increased invasion of epithelial PCa cells *in vitro*. Proteome Profiler Analysis revealed that monocyte‐derived CM from metastatic castration‐resistant (mCRPC) patients presented high levels of chitinase‐3‐like 1 (CHI3L1, YKL‐40) when compared to patients with stable disease (PCa‐N) and healthy control individuals (HC). The only described receptor for CHI3L1, interleukin‐13 receptor α2 (IL‐13Rα2), was significantly up‐regulated in the human metastatic PCa cell line, ARCaP_M_. Accordingly, we observed that the activation of IL‐13Rα2 from PCa‐M CM increased the invasiveness of ARCaP_M_ cells while siRNA directed against this receptor significantly reduced invasiveness of these cells in the presence of CM from PCa‐M patients.

**Conclusions:**

Thus, we show that circulating monocytes from metastatic PCa patients exert a tumor‐promoting role via the secretion of CHI3L1, and CHI3L1 demands further exploration as a possible therapeutic target in advanced PCa.

## 1 INTRODUCTION

Prostate cancer (PCa) is the most common cancer affecting men in the USA.^1^ It is the third leading cause of cancer death for men in America and remains an important public health concern. As PCa progresses, it can evolve into metastatic castration‐resistant PCa (mCRPC), a more lethal form marked by resistance to androgen deprivation therapy (ADT). The potential for immunotherapy directed against PCa is illustrated by sipuleucel‐T (Provenge; the first modern immunotherapy approved for mCRPC), where Kantoff et al^2^ showed a 22% reduction in death and minimal toxicity associated with its use. Accordingly, approval of sipuleucel‐T remains an important milestone for the field of PCa. There was, however, little benefit observed in the regression of disease as assessed by serum prostate‐specific antigen (PSA) or radiographs. These findings have left the field with many questions, but they have also clearly demonstrated that there is a potential role for immunotherapy in PCa. Approved modern immunotherapies, such as immune checkpoint inhibitors, have made their indelible mark on other solid‐tumor cancers such as melanoma, lung cancer, and kidney cancer. Deeper investigation into the role of the human immune system in PCa is vital in order to harness this kind of immune potential directed at future PCa therapies.

Monocytes are an essential component of the circulating immune system; these cells are dynamic and respond to foreign information in the bloodstream by producing growth factors and cytokines. Monocytes comprise most of the infiltrating cells associated with solid tumors.^3^, ^4^ Their recruitment and activation at tumor sites are largely regulated by tumor‐derived signals including chemokines, cytokines, and other endogenous signals.^3^ Following their recruitment into tumor tissues, monocytes can differentiate into tumor‐associated macrophages (TAMs), a diverse population in both phenotype and function. As key components of the PCa tumor microenvironment, macrophages have been shown to exert a protumorigenic effect by facilitating tumor cell migration and invasion.^5^ Our current knowledge, however, remains very limited regarding a plausible link between monocytes, macrophages, and PCa progression. Consequently, a more comprehensive understanding of the cellular interactions that contribute to PCa progression is necessary to support the development of effective immunotherapeutic strategies.

In the present study, we tested the hypothesis that circulating monocytes from advanced PCa patients directly influence the tumor microenvironment in distant organs such as the liver, lung, and bones and have a tumor‐promoting phenotype. We found that monocytes from advanced PCa/mCRPC (PCa‐M) secreted significantly higher levels of IL‐1β and chitinase‐3‐like 1 (CHI3L1) compared with monocytes from those with stable disease (PCa‐N) which narrowed our focus to these two prospective proteins. Previous research has described an IL1‐IL1R‐dependent mechanism as being essential in shaping the tumor‐promoting phenotype of monocytes and macrophages.^6^ Additionally, macrophage‐derived IL‐1β has been shown to influence the growth and regulation of androgen receptor (AR) and PSA levels in androgen‐responsive prostatic epithelial cells (LNCaP).^7^ The expression of CHI3L1 has been associated with a poor prognosis in PCa. Jeet et al^8^ reported that some PCa cell lines synthesize CHI3L1, a chitinase‐like protein, and this expression has been implicated in driving both migration and invasion of these cancer cells.

Herein, our results suggest that monocyte‐produced IL‐1β is not responsible for the protumor phenotype of monocytes from PCa patients. However, we demonstrate that CHI3L1 plays an IL‐13Rα2‐dependent role in the invasiveness and motility of PCa cell lines in culture. Collectively, these findings are the first to demonstrate a critical role for circulating monocytes in the progression of PCa.

## 2 METHODS

### 2.1 Patients and samples

Blood specimens were collected and studied under Cedars‐Sinai Medical Center Institutional Review Board approved protocols. Patient samples were classified into two categories: nonmetastatic (PCa‐N) and metastatic (PCa‐M). Healthy controls (HC) were classified as having no known history of cancer. The provided Table S1 indicates clinical characteristics of each patient sample used in this study.

### 2.2 Isolation and culture of human monocytes

Peripheral blood mononuclear cells (PBMC) were obtained using standard Ficoll gradient (GE Healthcare, Waukesha, WI, USA). Monocytes were isolated using anti‐CD14 magnetic beads (STEMCELL Technologies, Cambridge, MA, USA) and plated (0.3 × 10^6^) in 500 μL OPTI‐MEM containing 0.5% FBS, 25 U/mL penicillin, and 25 U/mL streptomycin in a 24‐well plate and incubated overnight at 37°C. Conditioned media (CM) were collected and centrifuged, and cell‐free supernatants were used for ELISA, Proteome Profiler Analysis, invasion, and growth assays (see below). Flow cytometry analysis classified circulating monocytes from PCa‐M, PCa‐N, and HC as classical (CD14^high^CD16^−^) and nonclassical (CD14^mid^CD16^high^). Antibodies were purchased from Biolegend (San Diego, CA, USA) and eBioscience (San Diego, CA, USA).

Alternatively, monocytes were differentiated into macrophages by culturing in RPMI 1640 medium containing 10% FBS, 50 IU/mL penicillin, 50 μg/mL streptomycin, and 20 ng/mL M‐CSF (R&D Systems, Minneapolis, MN, USA). On day 3, fresh media containing growth factors were added. At day 7, cells were stimulated for 3 hours with 1 μg/mL LPS‐EB Ultrapure (*E. coli* 0111: B4; InvivoGen, San Diego, CA, USA) +5 mM ATP (last 1 hour of stimulation; Sigma‐Aldrich, St. Louis, MO, USA). CM were collected and centrifuged, and cell‐free supernatants used for ELISA and/or invasion assays. Caspase‐1 activation was detected by flow cytometry using FLICA assay (ImmunoChemistry Technologies, Bloomington, MN, USA) according to manufacturer's instructions.

### 2.3 Tumor cell invasion and migration assays

For invasion, cells were fasted in 0.1% BSA and then plated (0.1 × 10^6^) on transwell inserts (8 μm) coated with matrigel matrix, phenol red free (BD Biosciences, Franklin Lakes, NJ, USA). Inserts were then placed in 24‐well plates containing 500 μL cell‐free monocyte‐CM (50% v/v) from PCa‐N, PCa‐M, or HC and incubated at 37°C for 48 hours. Alternatively, inserts were placed in 24‐well plates containing 20 ng/mL rhIL1β or 10 μM rhCHI3L1. Post‐incubation, media were aspirated and noninvaded cells on upper side of membrane were removed with a swab. Cells attached to the bottom side of membrane were fixed with 4% paraformaldehyde and stained with 0.1% (v/v) crystal violet. Inserts were washed and photographed at 10× using an inverted microscope (Leica, Wetzlar, Germany) and MagnaFire‐SP software. Migration assays were conducted using Incucyte^®^ Zoom Live Cell Analysis System (IncuCyte, Ann Arbor, MI, USA.). Cells were grown to confluence in matrigel‐coated 96 wells plates (IncuCyte, Ann Arbor, MI, USA). After overnight fasting (0.1% BSA T‐medium), a scratch was made using a 96‐pin WoundMaker (IncuCyte, Ann Arbor, MI, USA) and monocyte‐CM (50% v/v) from PCa‐N, PCa‐M, or HC or increasing concentrations of rhIL1β (0.2, 2, 20, and 100 ng/mL) was added. Cells were automatically imaged every hour. The data were analyzed using an integrated relative wound density protocol as previously described and recommended by the manufacturer (IncuCyte, Ann Arbor, MI, USA). Fetal bovine serum (FBS; 10%) and rhHGF (50 ng/mL) were utilized as positive controls for cell migration in ARCaP_M_ and PC3 cells,^9^ respectively.

### 2.4 Short‐interfering RNA (siRNA) targeting of IL‐13Rα2 in PCa cell line

ARCaP_M_ cells were transfected with 40 pmol of IL‐13Rα2‐specific (pool of 3 target‐specific 19‐25 nt) or nontargeting control siRNAs (Santa Cruz Biotechnology, Dallas, TX, USA) according to manufacturer's instructions. At 24 hours posttransfection, cells were fasted overnight in 0.1% BSA, harvested, and used for invasion assays.

### 2.5 Cells and culture conditions

ARCaP_M_ and C4‐2 cells used for this study were provided by Dr. Leland Chung. PC3 cells were provided by Dr. Carrie Rinker‐Schaeffer. LNCaP and 22Rv1 cells were purchased from ATCC. ARCaP_M_ cells were cultured in T‐medium (GibcoBRL, Grand Island, NY, USA) supplemented with 5% heat‐inactivated FBS (Omega Scientific, Inc, Tarzana, CA, USA). PC3, C4‐2, and 22Rv1 were cultured in RPMI 1640 with 10% FBS. LNCaP cells were cultured in RPMI 1640 with 10% FBS supplemented with 4.5 g/L glucose. Each had 50 IU/mL penicillin and 50 μg/mL streptomycin (GibcoBRL) and was maintained in 5% CO_2_ at 37°C. All cells were negative for mycoplasma contamination (MycoAlert Mycoplasma Detection Kit, Lonza, Walkersville, MD, USA). Cell line authentications were verified via short tandem repeat analysis using a DNA collection kit (DDC Medical, Fairfield, OH, USA).

### 2.6 MTT assay

In vitro cell proliferation was assessed using MTT assay as indicated by the manufacturer (ThermoFisher, Waltham, MA, USA) at day 6 of culture.

### 2.7 Cytokine and chemokine array

Protein array (Proteome Profiler™ Human XL Cytokine Array Kit, R&D, Minneapolis, MN, USA) surveyed 102 proteins in monocyte‐CM (50%v/v) of PCa‐N, PCa‐M, and HC according to manufacturer's instructions. The membrane was exposed to X‐ray film for 300 seconds, and profiles of mean spot pixel density were measured using Western Vision Software specific for R&D array analysis.

### 2.8 ELISA assay for chitinase‐3‐like 1 and IL‐1β

Proteins were assessed in cell‐free monocyte supernatants (CM) using chitinase‐3‐like 1 Quantikine ELISA and IL‐1β/IL1‐F2 Duo Set ELISA (R&D Systems) according to manufacturer's instructions.

### 2.9 Quantitative PCR

ARCaP_M_, PC3, 22Rv1, and LNCaP cells were lysed in Trizol (Life Technologies, Invitrogen, Carlsbad, CA, USA), and total RNA was prepared using RNAeasy kit (Qiagen, Germantown, MD, USA) according to manufacturer's instructions. 500 ng of total RNA was reverse‐transcribed and cDNA used for quantitative qPCR analysis on QuantiStudio5 Real‐Time PCR system (Applied Biosystems, Foster City, CA, USA) per manufacturer's instructions. In all cases, target gene expression was normalized to the expression of the housekeeping gene, 18S or GAPDH. Relative gene expression was calculated using standard 2‐∆∆Ct. IL13RA2 (Hs00152924_m1) and CHI3L1/YKL40 (Hs01072228_m1) were purchased from Applied Biosystems. IL1R1 (F: GTGGTATAAGGATTGCAAACCTC; R: ACATTCATCACGATGAGCCT and IL1R2 (F: CGTCTGCACTACTAGAAATGC; R: GCAGGAAAGCATCTGTATTCTC) were purchase via IDT Technologies, Coralville, IA, USA.

### 2.10 Statistical analysis

Statistical significance was calculated by Student's *t* test when comparing two groups or by one‐way ANOVA when comparing three or more groups. A *P*‐value of ≤0.05 was considered statistically significant.

## 3 RESULTS

### 3.1 Monocyte‐derived conditioned media from metastatic PCa/mCRPC patients promote PCa cell invasion and migration

**Figure 1:**
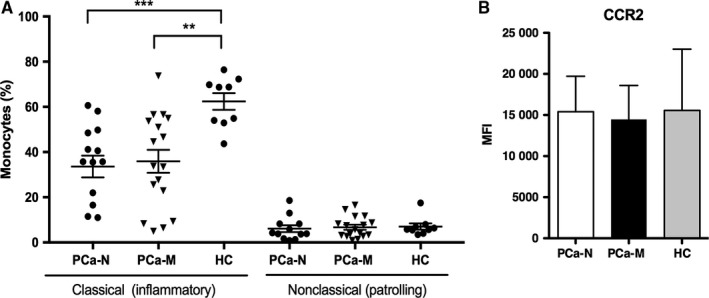
Characterization of monocyte population in PCa patients. A, Percentage of CD14^+^CD16^−^ (classical) and CD14^+^CD16^+^ (nonclassical) populations were analyzed by flow cytometry in PBMC from PCa‐M and PCa‐N and HC. B, Classical monocytes from PCa‐M (n = 12), PCa‐N (n = 14), and HC (n = 8) were analyzed for the expression of CCR2 (MFI, mean intensity of expression)

Monocytes from PCa‐M and PCa‐N were analyzed for markers that designate classical “inflammatory” and nonclassical “patrolling.” Flow cytometry showed a similar percentage of classical monocytes in both groups of PCa patient (35.9 ± 5.0% in PCa‐M n = 17 and 33.6 ± 4.8% in PCa‐N n = 14). Furthermore, when compared to HC blood samples, PCa patients had significantly less inflammatory monocytes (Figure 1A). Because CCR2 expression has been described as a marker for inflammatory monocytes and its expression is associated with progression of human breast cancer,^10^ we analyzed the expression of CCR2. In all patients analyzed, approximately 95% of the inflammatory monocytes were CCR2^+^ and there was no difference in CCR2 expression levels between PCa patients and HC individuals (Figure 1B).

**Figure 2:**
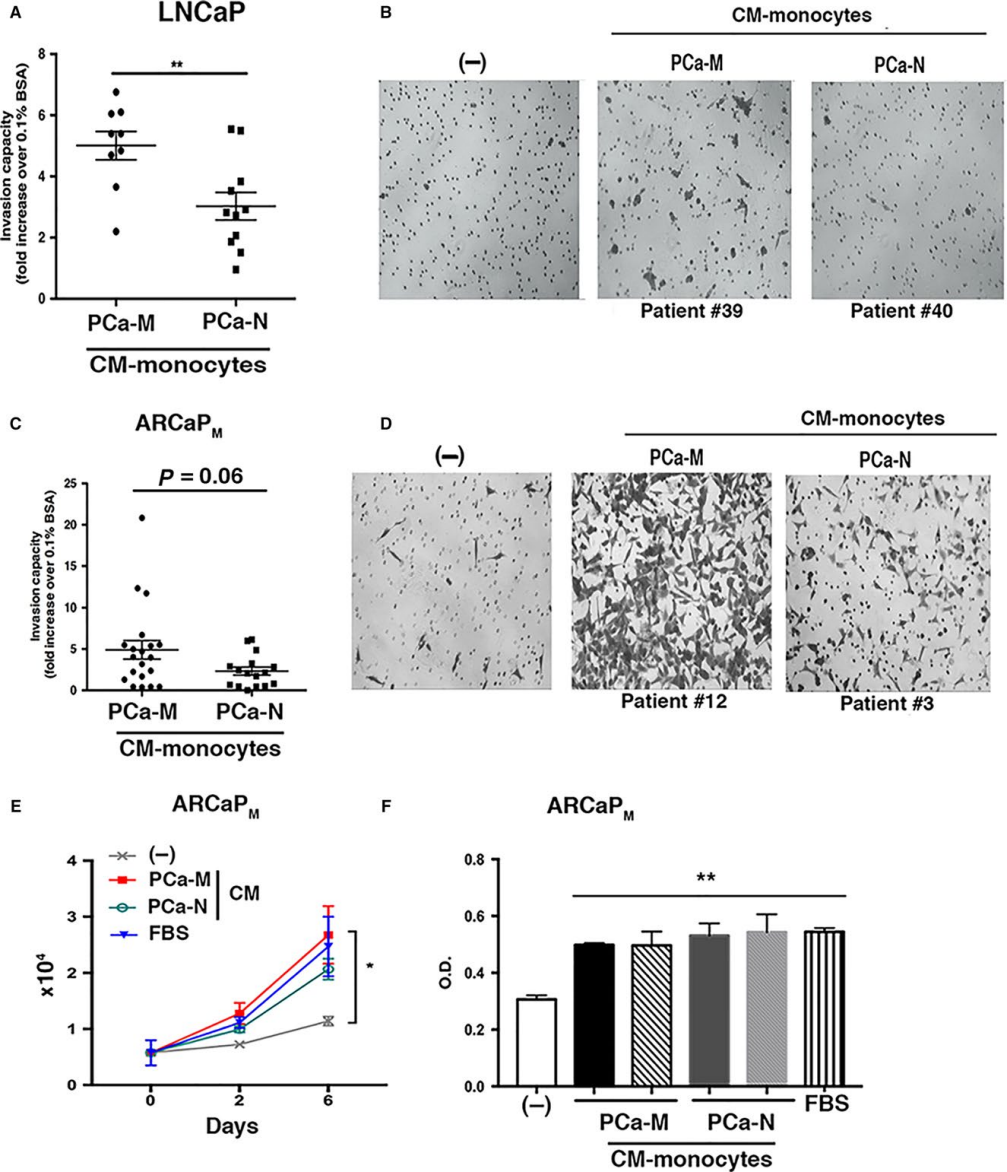
Monocyte‐CM or macrophage‐CM derived from metastatic PCa patients enhanced the invasion of tumor cells in vitro. Matrigel invasion assay of (A and B) LNCaP and (C and D) ARCaP_M_ at 48 h postincubation with monocyte‐CM as indicated. A, (LNCaP cells) shows each patient mean ± SEM of cells counted in 4‐5 representative microscopic fields per membrane (PCa‐M: n = 9; PCa‐N: n = 11) **P < 0.01. C, (ARCaP_M_ cells) shows each patient mean ± SEM of cells counted in 4‐5 representative microscope fields per membrane and analyzed using ImageJ software (PCa‐M: n = 20; PCa‐N: n = 16) *P* = 0.06. E, 1 × 10^4^ ARCaP_M_ cells cultured in 96‐well plate. Eighteen hours later, cells were fasted for 3 h and then cultured with monocyte‐CM from PCa‐M, PCa‐N, 10% FBS, or fasting media. Viable cells were counted at days 0, 2, and 6. Error bars represent mean ± SEM of three replicates per day. F, ARCaP_M_ cells were cultured under identical conditions described in (E) above, and growth was analyzed by MTT assay. Data are representative of three independent experiments (PCa‐M: n =* *7; PCa‐N: n =* *5). *P < 0.05; **P < 0.01 vs fasting media (‐)

We next examined whether secreted proteins from PCa monocytes or macrophages influenced the metastatic behavior of PCa cells via *in vitro* invasion assays using LNCaP (castration‐sensitive, low metastatic potential) and ARCaP_M_ (castration‐resistant, high metastatic potential) cells. We found that LNCaP cells exposed to monocyte‐CM from PCa‐M became significantly more invasive than when exposed to monocyte‐CM from PCa‐N (Figure 2A,B). We also observed that ARCaP_M_ cells tended to be more invasive when exposed to PCa‐M monocyte‐CM relative to cells exposed to CM from PCa‐N (Figure 2C,D), though in this sample these differences did not reach statistical significance. Macrophage‐CM from PCa‐M also increased the invasiveness of PC3 cells (Figure S1A) and C4‐2 cells (Figure S1A). Conversely, macrophage‐CM from PCa‐N reduced the invasion of these PCa cell lines.

Next, we analyzed whether monocyte‐CM from PCa patients affected growth of ARCaP_M_ cells. CM from PCa‐M and PCa‐N increased the growth of ARCaP_M_ cells similarly to that observed when exposed to media containing 5% FBS (Figure 2E, absolute numbers and Figure 2F, MTT assay). However, there was no difference in cell growth mediated by monocyte‐CM from patients with aggressive or localized disease.

**Figure 3:**
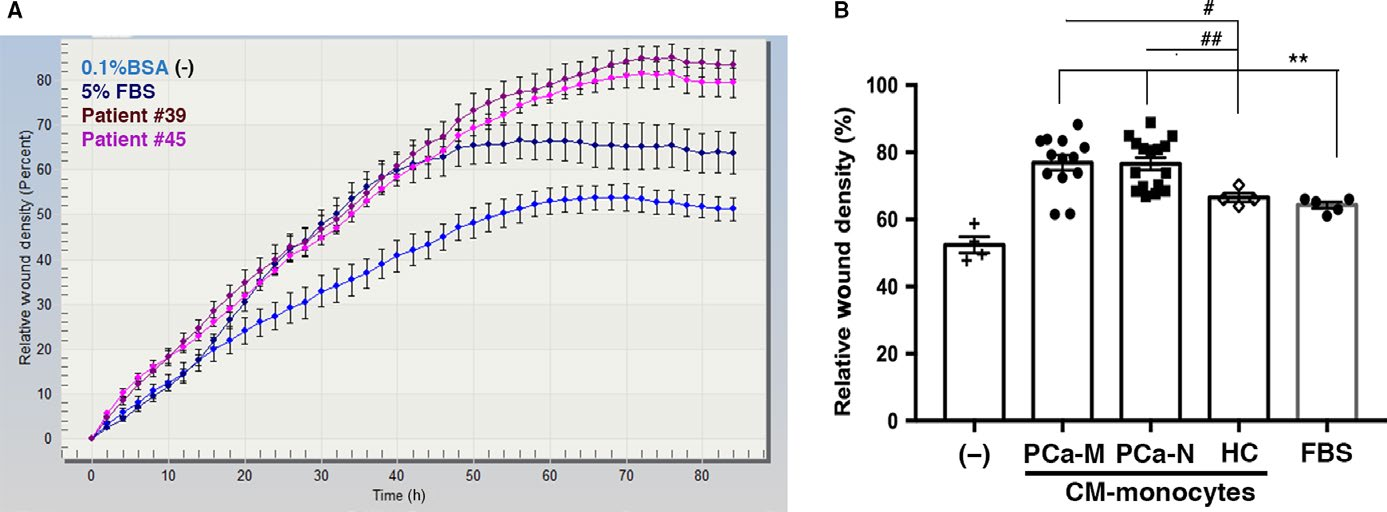
Monocyte‐CM from PCa patients enhanced the motility of tumor cells. A, 5 × 10^4^ ARCaP_M_ cells were plated in matrigel‐coated 96‐well plates. After overnight fasting, a wound was made in the cell layer and cells were exposed to either 0.1% BSA (fasting media), monocyte‐CM from PCa‐M (n = 13), PCa‐N (n = 16), HC (n = 4), or 5% FBS. All samples were performed in triplicate. Scratched field images were obtained using IncuCyte Live Cell Imaging System, and graphs indicate percentage of relative wound density followed for 72 h from two independent experiments. B, Quantitative analysis of tumor cell motility. Data are mean ± SEM of triplicate wells. PCa‐M vs HC #*P* < 0.05; PCa‐N vs HC ##*P* < 0.01; PCa‐M and PCa‐N vs FBS **P < 0.001; PCa‐M and PCa‐N vs fasting media ***P < 0.0001

Wound‐healing assays were performed using real‐time quantitative cell analysis (Incucyte ZOOM System) to determine the effect of monocyte‐CM from PCa patients on ARCaP_M_ cell motility. CM‐monocytes from PCa‐M and PCa‐N markedly increased the motility of these cells in a wound‐healing assay when compared with CM‐monocytes derived from healthy controls (HC), fasting media or 5% FBS. (Figure 3A,B, Figure S2).

### 3.2 Monocyte‐derived conditioned media from metastatic PCa patients contain high levels of IL‐1β and CHI3L1

**Figure 4:**
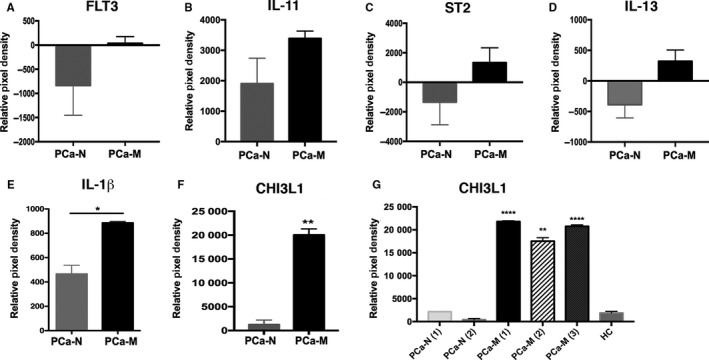
CHI3L1 and IL‐1β are the most abundant proteins secreted by monocytes from metastatic PCa patients. Proteomic profile analysis of monocyte‐CM from PCa‐M (n =* *3), PCa‐N (n =* *2), and HC (n =* *1) collected after 24 h of culture. A‐F, Proteins that were up‐regulated or down‐regulated and reproducible in the patient samples analyzed. **P < 0.01 when PCa‐M compared with PCa‐N. G, Levels of CHI3L1 analyzed in duplicate. Data are mean ± SEM from each patient or healthy control analyzed in duplicate. ***P < 0.001; ****P < 0.0001 when PCa‐M was compared with PCa‐N or HC

To identify potential proteins secreted by monocytes from PCa‐M that might mediate invasiveness of PCa tumor cells, we performed a proteomic profiling analysis of 102 cytokines and chemokines. We compared profiles across CM obtained from PCa‐M, PCa‐N, and HC. In our analysis, we excluded proteins that were not reproducible among all patients within a category, and any proteins that were coexpressed by PCa and HC. FLT3 (Figure 4A), IL‐11 (Figure 4B), ST2 (Figure 4C), IL‐13 (Figure 4D), IL‐1β (Figure 4E), and CHI3L1 (Figure 4F,G) were up‐regulated in monocyte‐CM from PCa‐M but not from PCa‐N and HC. Moreover, both CHI3L1 and IL‐1β displayed statistically significant up‐regulation in PCa‐M CM compared with that of PCa‐N or HC.

### 3.3 IL‐1β signaling is not responsible for the protumor phenotype of monocytes from PCa‐M patients

When stimulated with LPS+ATP (triggering the inflammasome pathway), we observed that monocyte‐derived macrophages from PCa‐M patients secreted higher levels of IL‐1β (Figure S3A). These results and our proteomic analysis led us to hypothesize that monocyte‐produced IL‐1β from PCa‐M could be, at least in part, responsible for promoting the invasive behavior of PCa cells. To address this, we analyzed the percentage of FLICA expression as an indicator of caspase‐1 activation after stimulating macrophages with LPS+ATP. Our results showed no significant differences in caspase‐1 activation between PCa‐N and PCa‐M (Figure S3B).

**Figure 5:**
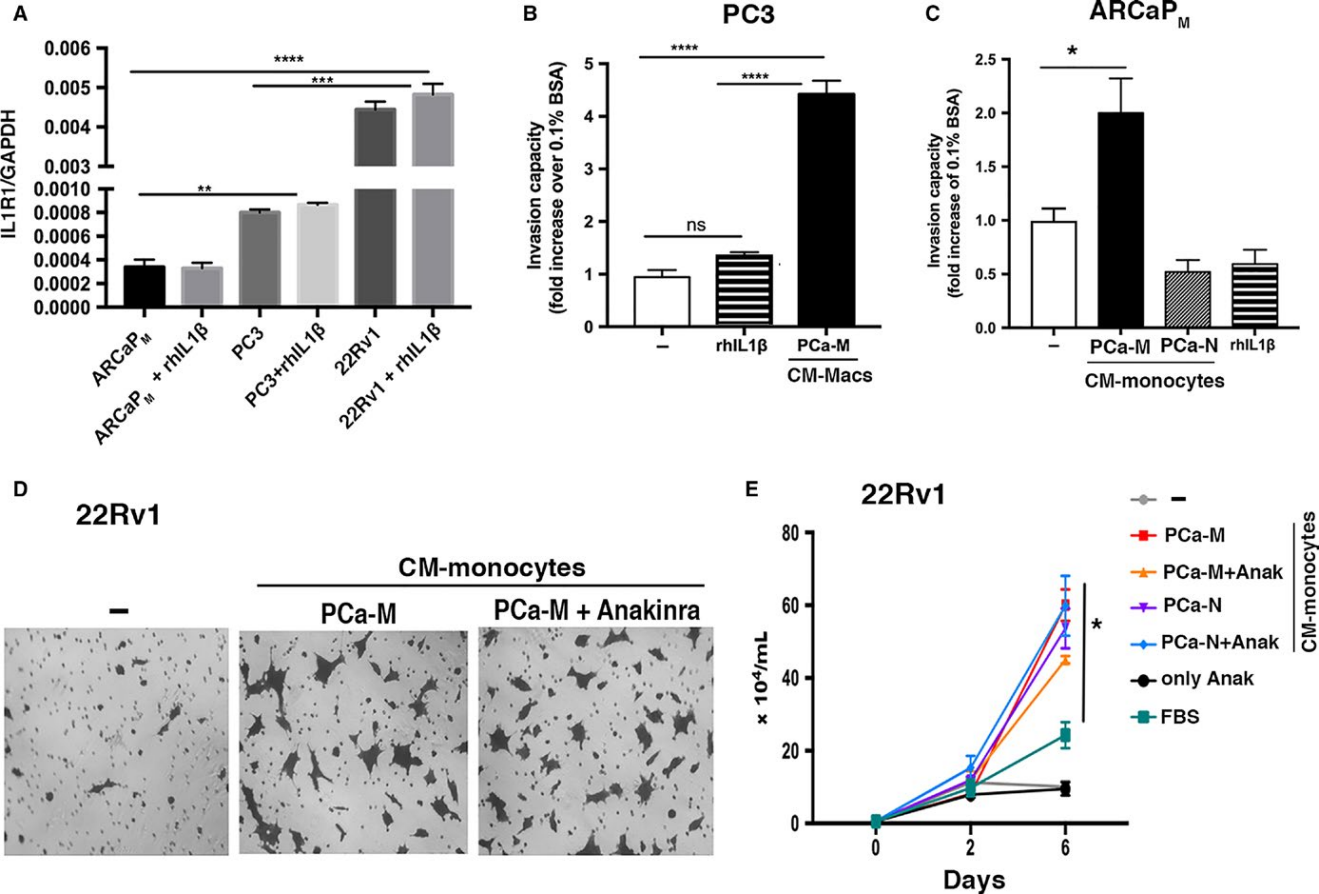
IL‐1β is not the main factor present in monocyte‐CM that drives PCa cell invasiveness. A, ARCaP_M_, PC3, and 22Rv1 cells were cultured in fasting media (0.1% BSA) with or without 20 ng/mL of rhIL1β for 24 h. Expression of IL‐1R1 was analyzed by RT‐PCR. Data are mean ± SEM of two independent experiments performed in triplicate. B and C, Quantitative analysis of tumor cell invasion: 20 ng/mL rhIL1β did not enhance invasion of PC3 or ARCaP_M_ compared with monocyte‐CM from PCa‐M. Data are mean ± SEM and are representative of 2 or 3 independent experiments. D, Bright‐field images of 22Rv1 cell invasion at 48 h postincubation with monocyte‐CM from PCa‐M with or without Anakinra (500 ng/mL). E, Growth curve of 22Rv1 co‐cultured with monocyte‐CM from PCa‐M or PCa‐N, with or without Anakinra. *P < 0.05, **P < 0.01; ***P < 0.001; ****P < 0.0001. ns, nonstatistical significances. Comparisons indicated in graphs

Since it has been shown that IL‐1β activates androgen‐responsive prostatic epithelial cells,^7^ we analyzed the expression of IL‐1 receptor type I (IL‐1R1) mRNA in several PCa cell lines. We observed a strong expression of IL‐1R1 in androgen‐responsive 22Rv1, a moderate expression in androgen‐independent PC3, and low expression in androgen‐resistant ARCaP_M_. Additionally, the presence of recombinant human IL‐1β (rhIL1β) did not significantly affect the expression levels of IL‐1R1 in any of the three cell lines (Figure 5A). The expression of IL‐1R2 (decoy receptor) was elevated in PC3 and 22Rv1, but no expression was detected in ARCaP_M_ cells, independent of the presence of rhIL1β (Figure S4A). Furthermore, activation of AR with synthetic androgen (methyltrienolone, R1881) in 22Rv1 cells had no effect on the expression of IL‐1R1 (Figure S4B), suggesting that IL‐1β might affect 22Rv1 independently of AR activation.

Next, we directly compared the effects of PCa‐M CM vs rhIL1β on PCa cell invasiveness and motility. While PCa‐M CM induced invasion in PC3 and ARCaP_M_ cells, the presence of rhIL1β had no effect on invasion (Figure 5B,C) nor motility (Figure S5A,B) at any concentration (0.2, 20, and 100 ng/mL).

Finally, we assessed whether IL1‐ILR1 signaling is responsible for invasion of androgen‐responsive 22Rv1 cells in the presence of PCa‐M monocyte‐CM. While monocyte‐CM from PCa‐M augmented invasion of 22Rv1, the blockage of soluble IL‐1β via Anakinra had no effect on tumor cell invasion (Figure 5D) or growth (Figure 5E). Together, these findings suggest that IL1‐IL1R signaling is not the main pathway driving invasiveness of PCa cells.

### 3.4 CHI3L1 produced by monocyte‐CM from metastatic PCa patients modulates the invasion of tumor cells

**Figure 6:**
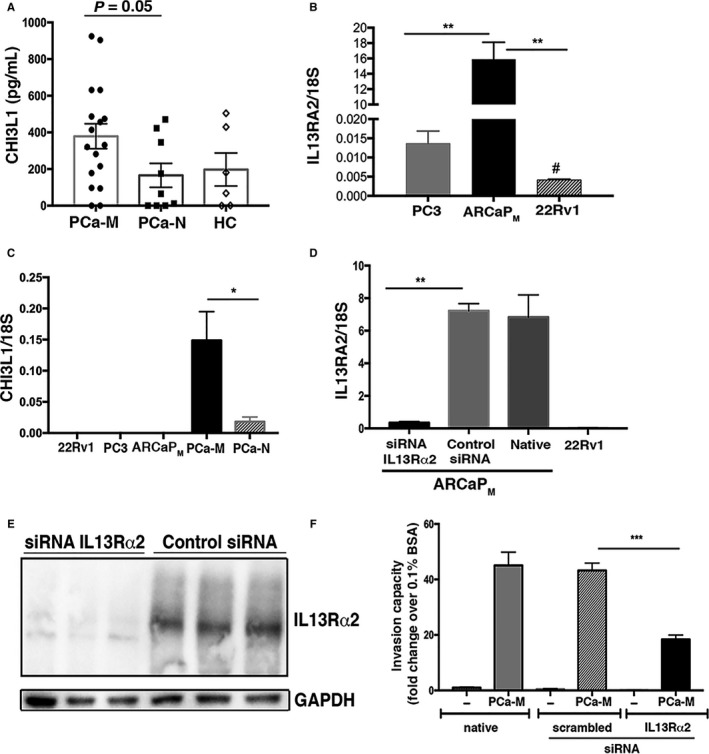
IL‐13Rα2 promotes invasion of PCa cells *in vitro* in response to monocyte‐CM from PCa‐M. (A) CHI3L1 validation: monocyte‐produced CHI3L1 was measured using Quantikine ELISA (PCa‐M n =* *17; PCa‐N n = 9; HC n =* *6). *P* = 0.05 (B) RT‐PCR of IL‐13Rα2 expression in PC3, ARCaP_M_, and 22Rv1. Data are mean ± SEM and representative of two independent experiments performed in triplicate. **P < 0.001 ARCaP_M_ cells compared to PC3 and 22Rv1; #*P* < 0.05 22Rv1 compared with PC3. C, TaqMan data showing CHI3L1 expression in PCa cell lines vs monocytes from PCa‐M and PCa‐N. Data are mean ± SEM from PCa‐M (n* *=* *6) and PCa‐N (n* *=* *5) patients. *P < 0.05 PCa‐M vs PCa‐N. (D) RT‐PCR and (E) immunoblot of IL‐13Rα2 expression in ARCaP_M_ cells confirming knockdown of receptor after siRNA approach. No expression of IL‐13Rα2 was detected in 22Rv1 cells. **P < 0.01 control (scrambled) siRNA vs IL‐13Rα2 siRNA or native cells vs IL‐13Rα2 siRNA. F, Invasion assay using ARCaP_M_ (native, scrambled siRNA or IL‐13Rα2 siRNA) at 48 h postincubation with monocyte‐CM from PCa‐M (n* *=* *7). Graphs show mean ± SEM of four representative microscope fields per membrane. ***P < 0.001 scrambled siRNA or native vs IL‐13Rα2 siRNA

Recently, it has been shown that CHI3L1 binds interleukin 13 receptor alpha‐2 (IL‐13Rα2) and that CHI3L1, IL‐13Rα2, and IL‐13 form a multimeric complex.^11^ To explore the role of CHI3L1 in PCa metastatic behavior, we first validated the previous cytokine array data by ELISA (Figure 6A). Next, we analyzed the expression of IL‐13Rα2 and observed that ARCaP_M_ cells expressed significantly higher transcript levels of IL‐13Rα2 compared with PC3, 22Rv1 (Figure 6B), and LNCaP cells (Figure S6A). Furthermore, IL‐13Rα2 transcripts were not detected in monocytes isolated from PCa‐M and PCa‐N (data not shown). Since it has been previously reported that PCa cell lines secrete CHI3L1,^8^ we tested the expression levels in 22Rv1, PC3, and ARCaP_M_ cells. TaqMan analysis showed little to no expression of CHI3L1 in any of the PCa cell lines tested (Figure 6C). Next, an siRNA knockdown approach was used to assess whether the CHI3L1/IL‐13Rα2 axis might be responsible for PCa tumor cell invasiveness driven by monocyte‐CM. IL‐13Rα2 knockdown was confirmed by qPCR (Figure 6D) and western blot (Figure 6E) analysis. Compared to control (scrambled) siRNA, IL‐13Rα2 siRNA significantly reduced the ability of PCa‐M monocyte‐CM to promote invasion of ARCaP_M_ cells (Figure 6F).

We next tested whether the binding of recombinant human CHI3L1 (rhCHI3L1) to IL‐13Rα2 could promote invasion of ARCaP_M_ cells. We found that 10 μM of rhCHI3L1 significantly increased the invasiveness of ARCaP_M_ and this effect was abrogated when IL‐13Rα2 was suppressed (Figure S6B). IL‐13 has been shown to bind with high affinity to IL‐13Rα2, thereby promoting invasion and metastasis of ovarian cancer cells.^12^ Because we found increased levels of IL‐13 in monocyte‐CM from PCa‐M (Figure 4D), we tested the effects of rhIL‐13 alone or combined with 10 nmol/L rhCHI3L1 in ARCaP_M_ invasiveness. There was no detectable effect of IL‐13 on the invasiveness of ARCaP_M_ cells in the presence or absence of IL‐13Rα2 expression (data not shown). Finally, we observed that the transcript expression of IL‐13Rα1, the primary receptor for IL‐13, in ARCaP_M_ cells was approximately 130‐fold lower than IL‐13Rα2 transcript levels (data not shown). Together, these findings support a role for monocyte‐derived CHI3L1 in modulating metastasis of PCa.

## 4 DISCUSSION

Development of metastatic disease is the hallmark of incurable PCa. Castration (medical or surgical) is used as a primary therapeutic maneuver in the management of patients with metastatic PCa. While initially effective, the disease eventually progresses to a resistant form, mCRPC. Through illness evolution, cancer spreads to the bone and other organs culminating in death. Metastasis itself is an intricate biological process characterized by invasion, intravasation, survival amid the circulating immune system, reinvasion, and durable growth at secondary sites. This elaborate process requires both evasion of immune responses and the generation of prometastatic signals including growth factors, cytokines, and chemokines.

Mouse models of spontaneous breast and skin cancers have revealed a role for myelomonocytic cells in driving malignant progression and metastasis.^13^, ^14^ These models have also shown that inflammatory monocytes, as precursors of TAMs, contribute to tumor metastasis and immunosuppression.^14^, ^15^ For example, Qian et al demonstrated that a distinct population of metastasis‐associated macrophages promotes the extravasation, seeding, and persistent growth of tumor cells. The authors defined the origin of these macrophages by showing that inflammatory monocytes are preferentially recruited to pulmonary metastases but not to primary mammary tumors in mice. This process holds true for human inflammatory monocytes in pulmonary metastases of human breast cancer cells.^14^ Inflammatory monocytes have also been shown to contribute to TAM accumulation and maintenance in a mouse mammary tumor model^16^ and to the establishment of pulmonary metastases derived from mouse or human breast cancer cells.^14^ While TAMs in the metastatic niches were not analyzed in the present study, we believe that the same described migration of inflammatory monocytes to metastatic sites likely occurs in PCa.

Previous studies have shown that the same tumor‐promoting functions and gene expression profile in monocytes from renal cell carcinoma (RCC) patients were mirrored in the TAMs isolated from RCC patients and human xenograft tumors.^6^ Prior reports addressing the role of myelomonocytic cells in tumor progression have focused mainly on macrophages, while the contributions of circulating blood monocytes in cancer development and progression remain largely unexplored. In the present study, we probed the hypothesis that secreted factors from monocytes alter the invasive properties of PCa cell lines. Specifically, we observed that monocytes from mCRPC promoted tumor invasiveness via an IL‐13Rα2‐dependent mechanism. These novel findings highlight a new pathway for PCa metastasis and underscore the importance of understanding the contributions of all immune cell types to PCa progression.

Human chitinase‐like glycoproteins are expressed in several types of solid tumors including breast, colon, kidney, SCLC, ovarian, prostate, and endometrial cancers as well as malignant melanoma, glioblastoma, and Hodgkin's lymphoma.^17^, ^18^ CHI3L1 is a chitinase‐like protein lacking enzymatic properties, and its mechanism of action remains an active area of exploration. The CHI3L1 protein was originally discovered in mouse breast cancer cells.^19^ More recently, this chitinase‐like protein has been directly associated with the progression and aggressiveness of PCa.^20^ Although not previously described as a secreted product of monocytes, we discovered that conditioned supernatants from PCa monocytes (and macrophages from the same patients; not shown) contain significant amounts of CHI3L1. Thus, our findings identify, for the first time, that circulating immune cells from advanced metastatic PCa patients secrete higher levels of CHI3L1, leading to increased invasion of tumor cells. Recently, CHI3L1 has been shown to bind the IL‐13Rα2 receptor expressed on gastric and breast cancer cells, thereby promoting metastasis of these tumor cells.^21^ These investigators showed that the binding of CHI3L1 to IL‐13Rα2 results in the recruitment of AP (activator protein)‐1 family members, which include several transcription factors important for tumor invasiveness as well as matrix metalloproteases. In our studies, we observed that CHI3L1, but not IL13, mediated IL‐13Rα2 activation which promoted tumor invasiveness, suggesting that these two ligands might promote differential signaling through IL‐13Rα2. Indeed, similar observations have been made with other cytokine ligand‐receptor interactions where it was observed that varying geometries, affinities, and/or duration of contact led to differential signaling.^22^, ^23^ Future studies are warranted to better characterize the signaling pathways activated by CHI3L1 in PCa cells.

**Figure 7:**
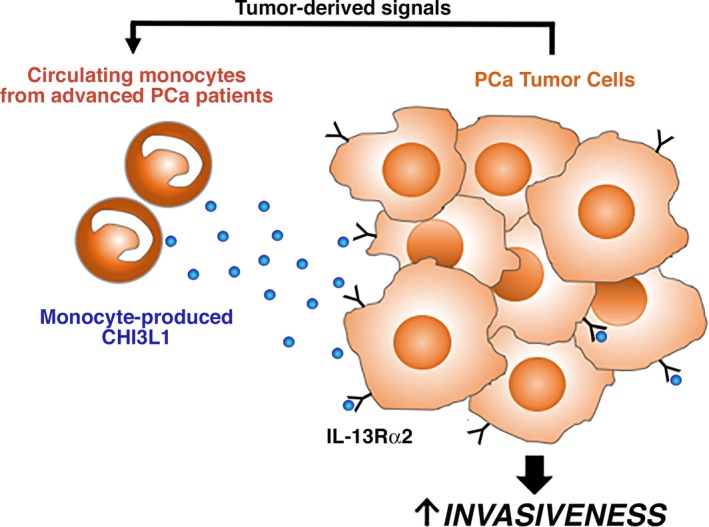
Circulating blood monocytes from advanced PCa patients produce CHI3L1 in response to tumor‐derived signals, thereby promoting tumor cell invasiveness

In summary, we have examined the mechanism by which a protein factor released from activated PCa monocytes contributes to the invasiveness and altered migration of PCa cells in functional in vitro assays. CHI3L1 may be a major factor that mediated these properties in PCa cells, and these effects were dependent on the expression of IL‐13Rα2 by PCa cells (Figure 7). With the continued development of therapeutics targeted toward manipulation of the immune response, altering the secretome from monocytes and macrophages is likely to emerge as an important therapeutic strategy and novel approach in cancer immunotherapy.

## CONFLICT OF INTEREST

The authors declare no conflict of interest.
