# Supplementary figures and images for: Circulating monocytes from prostate cancer patients promote invasion and motility of epithelial cells
Source: Cancer Med. 2018 Aug 9;7(9):4639–49. doi: 10.1002/cam4.1695 (PMC6143932; doi:10.1002/cam4.1695)

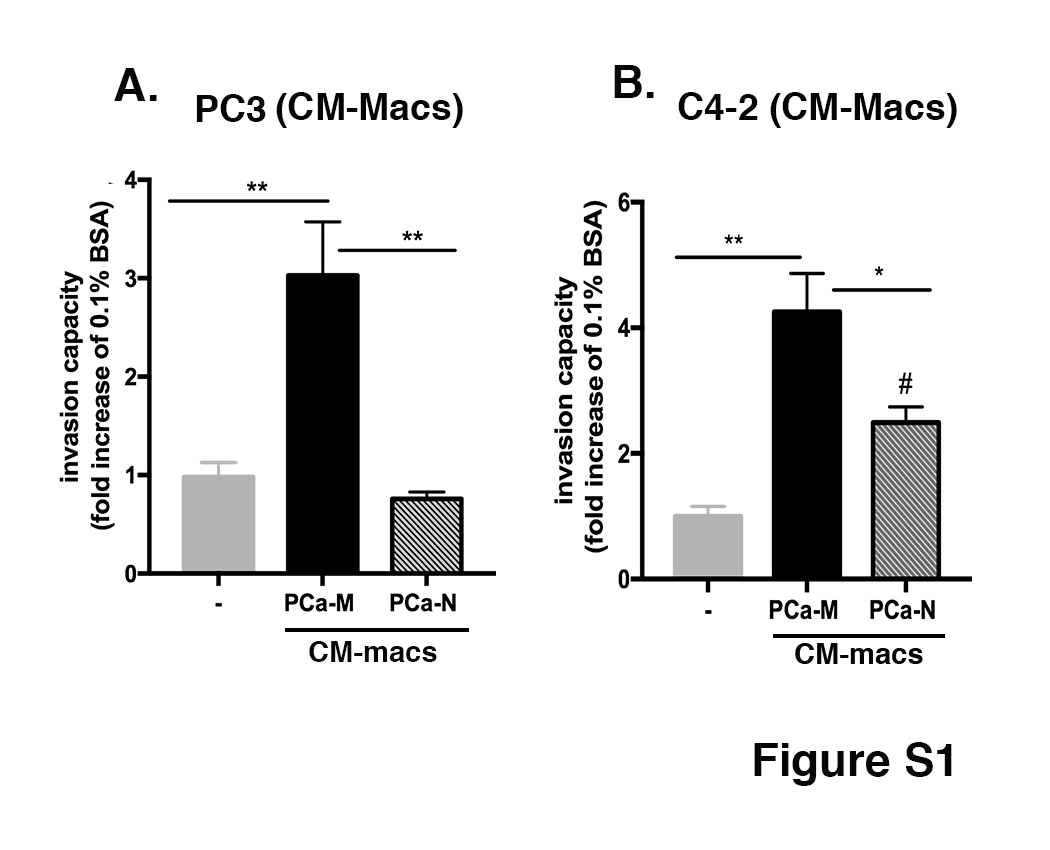

Supplement: Supplementary file 1 [file CAM4-7-4639-s001.tif]

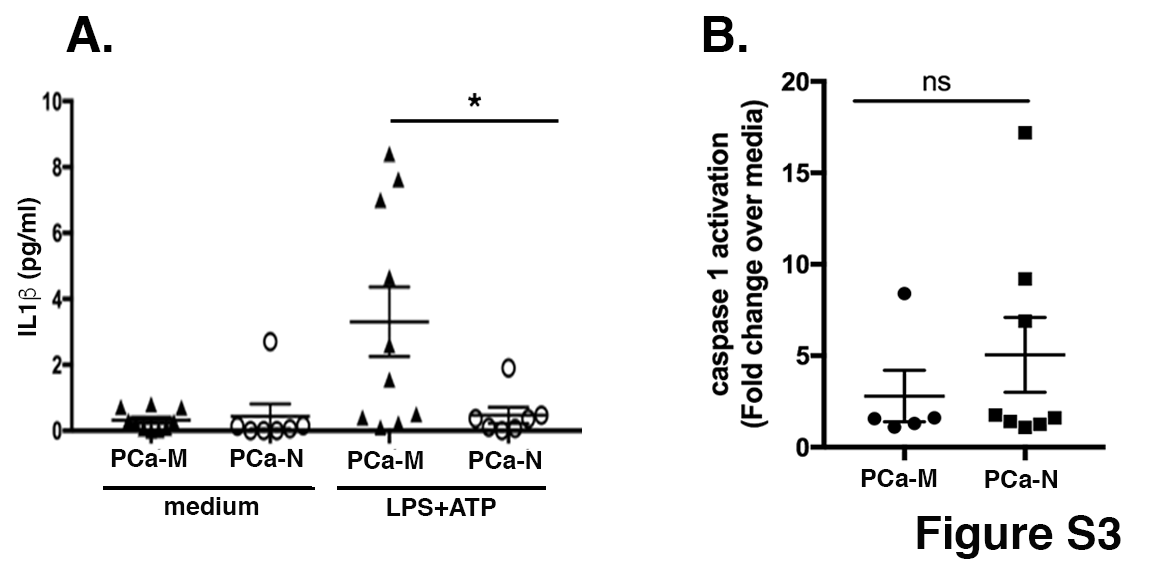

Supplement: Supplementary file 3 [file CAM4-7-4639-s003.tif]

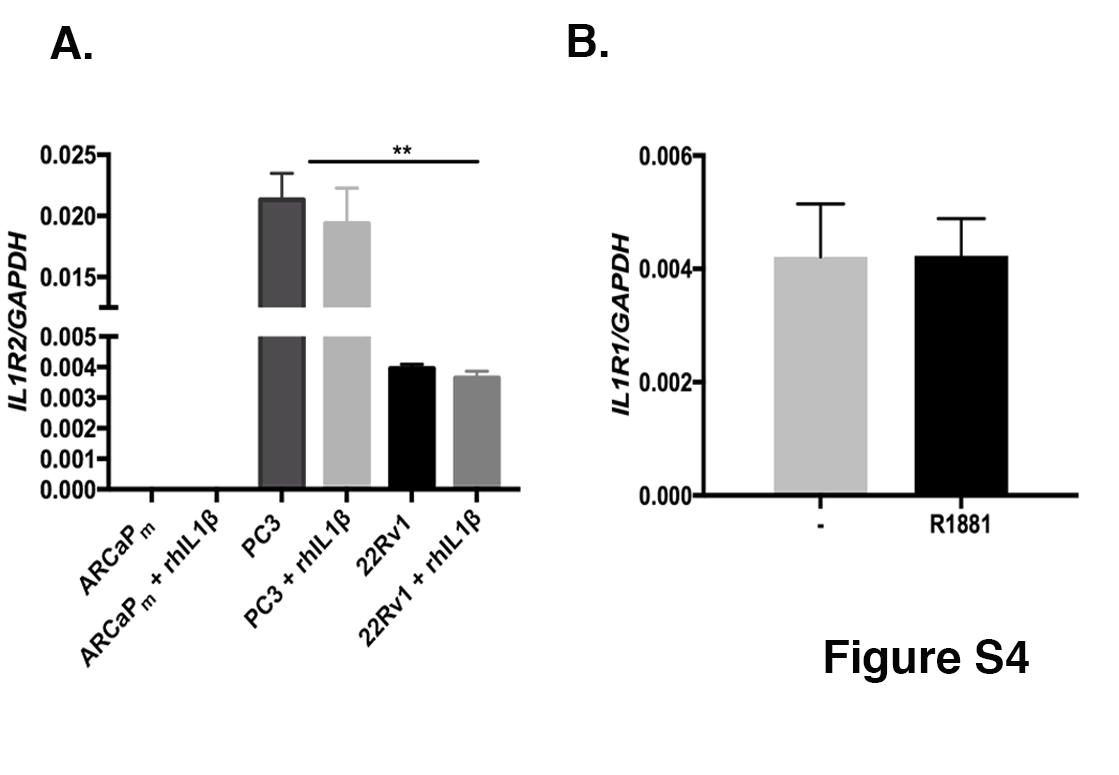

Supplement: Supplementary file 4 [file CAM4-7-4639-s004.tif]

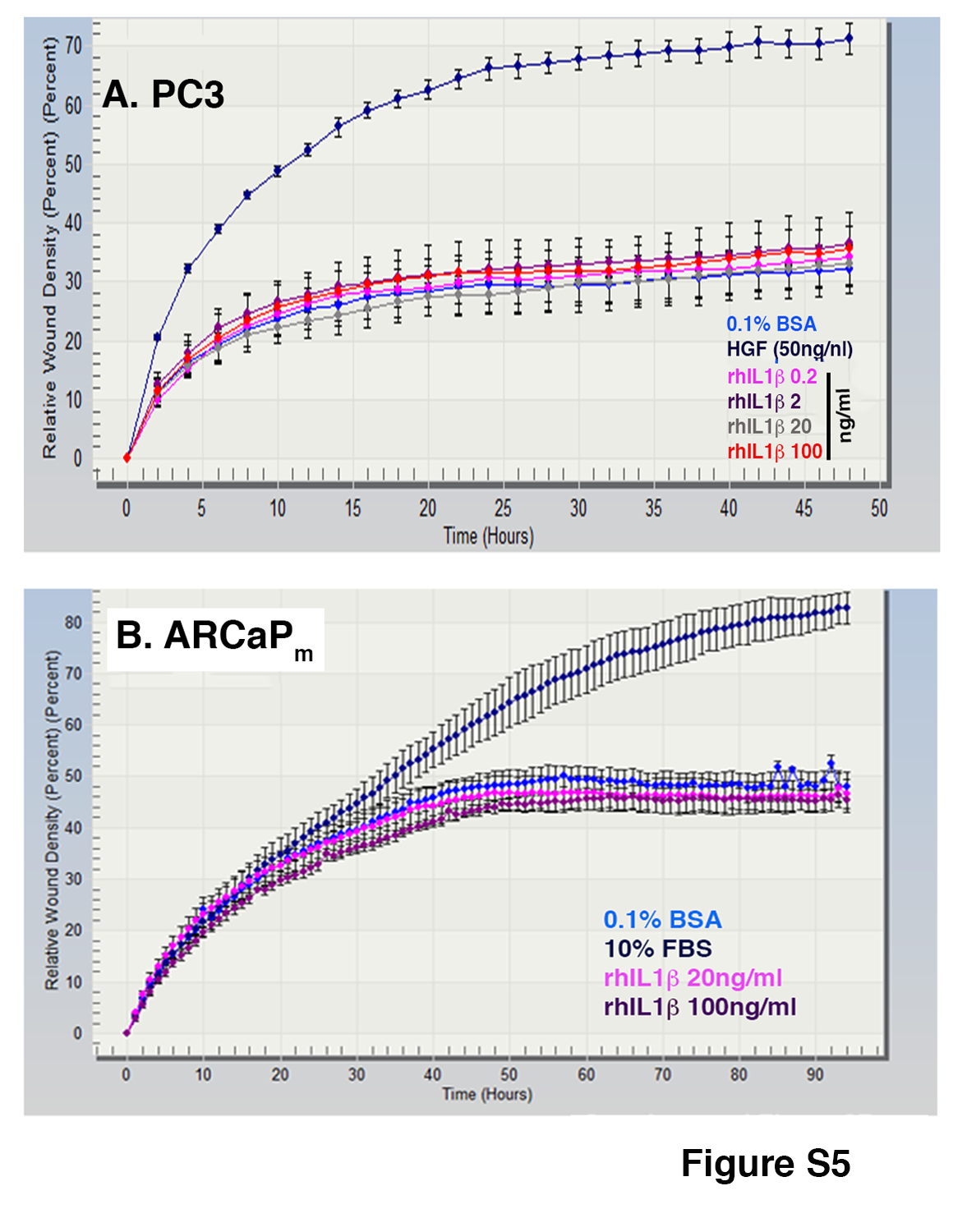

Supplement: Supplementary file 5 [file CAM4-7-4639-s005.tif]

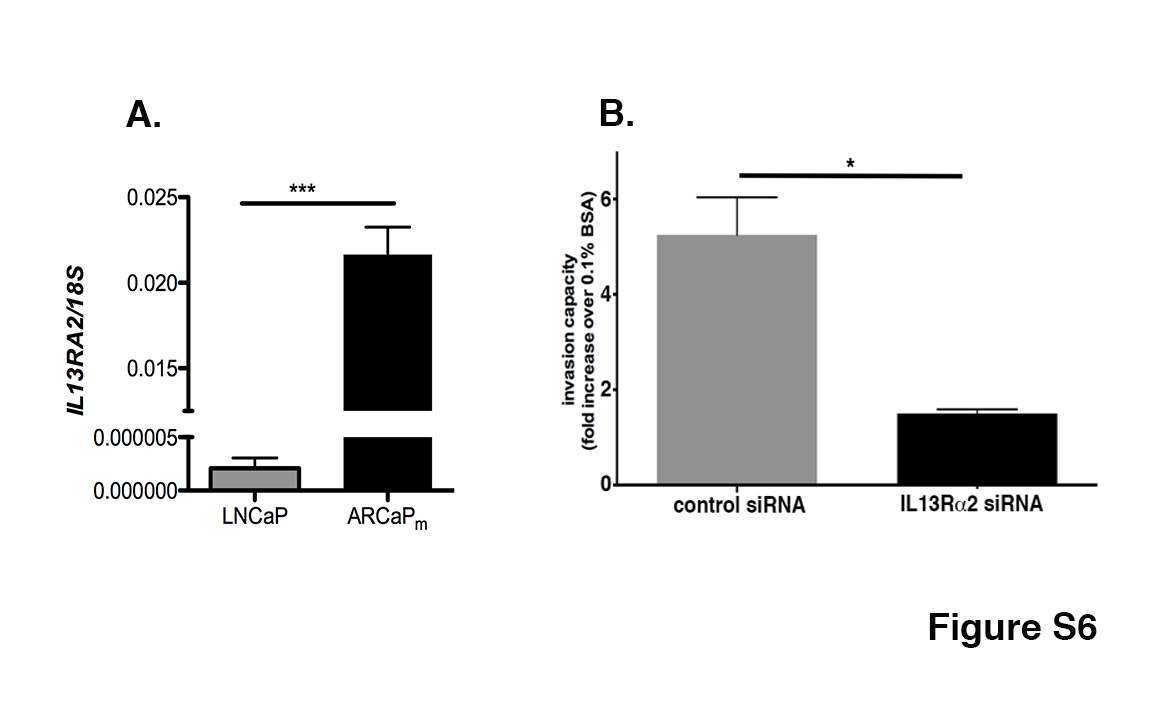

Supplement: Supplementary file 6 [file CAM4-7-4639-s006.tif]

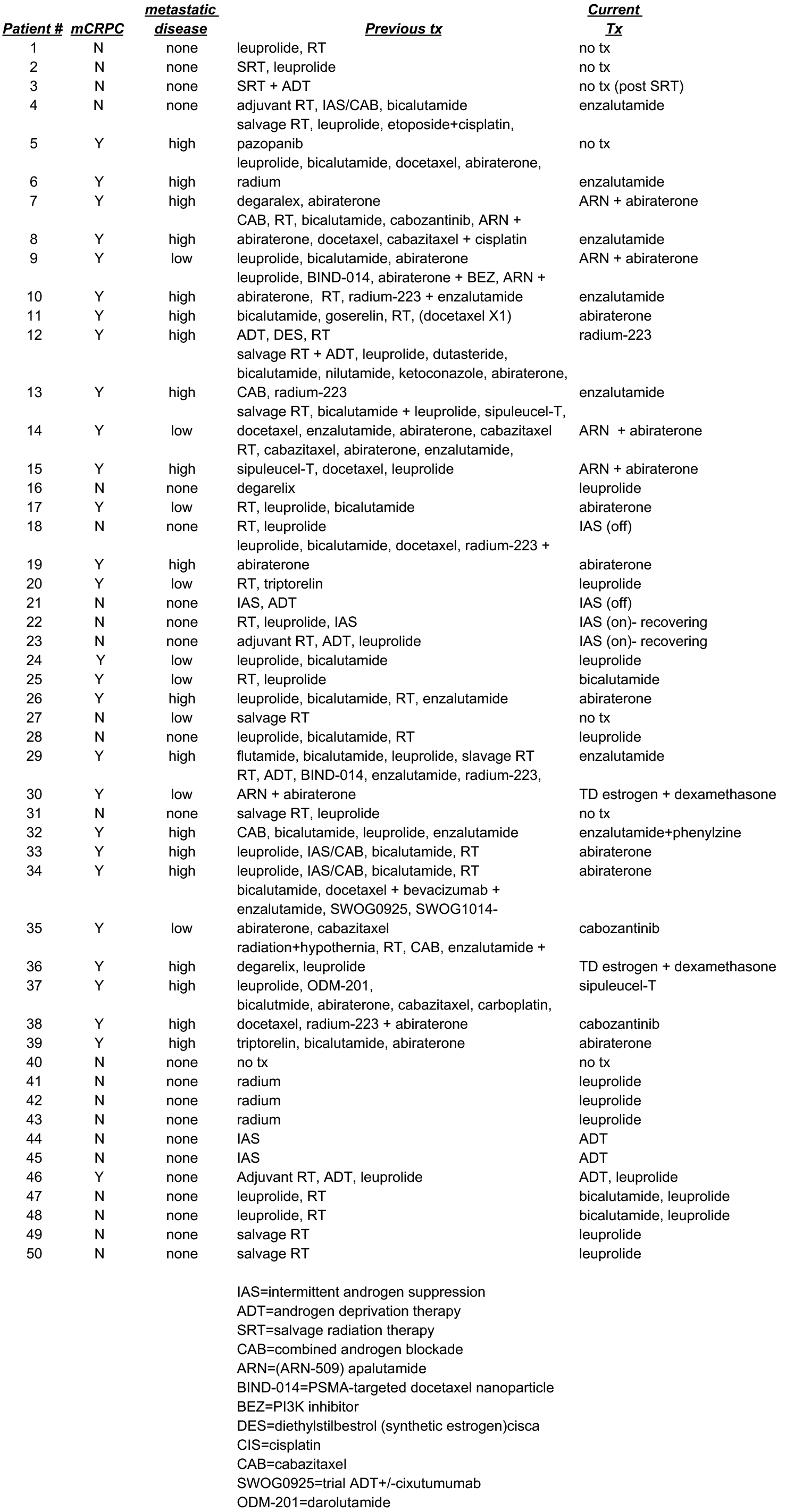

Supplement: Supplementary file 7 [file CAM4-7-4639-s007.tif]
